# Regional Disparity in Asthma Prevalence and Distribution of Asthma Education Programs in Texas

**DOI:** 10.1155/2020/9498124

**Published:** 2020-01-07

**Authors:** Jessica John, Juha Baek, Taehyun Roh, Lucia Cabrera-Conner, Genny Carrillo

**Affiliations:** ^1^Department of Health Promotion and Community Health Sciences, School of Public Health, Texas A&M University, 212 Adriance Lab Road, College Station, TX 77843, USA; ^2^Department of Environmental and Occupational Health, School of Public Health, Texas A&M University, 212 Adriance Lab Road, College Station, TX 77843, USA; ^3^Department of Epidemiology and Biostatistics, School of Public Health, Texas A&M University, 212 Adriance Lab Road, College Station, TX 77843, USA; ^4^Program on Asthma Research and Education, Healthy South Texas, Texas A&M School of Public Health, McAllen Campus, 2101 S. McColl Road, McAllen, TX 78503, USA

## Abstract

**Objectives:**

To identify the distribution of asthma education programs that are currently active in Texas and examine whether there is a geographical disparity between asthma prevalence and locations of asthma education programs in the Public Health Regions (PHRs) of Texas.

**Methods:**

The data for adult asthma prevalence in PHRs was obtained from the Texas Department of State and Health Services (DSHS) 2015 Texas Behavioral Risk Factor Surveillance System (BRFSS) Public Use Data File. The Geographic Information System (GIS) program was used to show the distribution of asthma education programs and visually identify the isolated areas for asthma education programs on the maps. To examine the areas covered by the asthma education programs, we illustrated 50 miles and 70 miles of buffer zones from each program by proximity (multiple ring buffer) functions in GIS.

**Results:**

We identified that 27 asthma education programs are active in Texas as of July 2019. The analysis showed that PHRs 1, 2, and 7 had the highest rate of asthma prevalence but had fewer asthma education programs. Also, the distribution of asthma education programs is concentrated around major cities, leading to a regional imbalance between asthma prevalence and locations of asthma education programs. The central and western areas of Texas proved to be marginalized areas for asthma education programs, particularly PHRs 2 and 9 because they may not be covered by the buffer zones of 70 miles from any asthma education programs. *Discussion*. This study revealed the marginalized regions in Texas lacking asthma education programs. The findings could help policymakers and health care professionals enhance opportunities to develop asthma education programs using different venues in isolated areas and prioritize these regions, for funds, to establish new asthma education programs.

## 1. Introduction

Asthma is a chronic medical condition in which a person's airways in the lungs become narrowed and swollen, which makes it difficult to breathe causing coughing, tightness in the chest, wheezing, and shortness of breath [1]. Currently, more than 25 million (approximately 7.7% adults and 8.4% children) people in the United States have asthma, with the disease being more common in boys (9.5%) than in girls (7.3%) [2, 3]. In adulthood, it reverses and more women (9.8%) than men (5.4%) have asthma [2, 3]. In particular, the prevalence of asthma in the US among adults in 2017 was 9.1%; 11.5% among females, 6.4% among males, 11.6% among African Americans, 9.3% among Whites, and 6.7% among Hispanics [4]. The Centers for Disease Control and Prevention (CDC) in 2017 reported that Texas had 7.3% and 7% of adults and children with current asthma, respectively [3].

Asthma has caused substantial burdens, both clinically and financially, and has become a significant health issue in the US. According to the CDC in 2017, there were 3,564 deaths attributed to asthma in the US, signifying that about 10 people per day lose their lives due to asthma [5]. In 2017, there were 232 deaths (8.3 death rate per million) in the state of Texas as per CDC reports [3]. Sullivan et al. reported that parents/caregivers of school-aged children (SAC) with asthma and an exacerbation missed 1.2 times more workdays (*p* < 0.05), while those with SAC with asthma and an Emergency Department/Inpatients visits missed 1.8 times more workdays (*p* < 0.01) than the parents of SAC without asthma [6]. However, a study using the 2008 Medical Expenditures Panel Survey to associate between asthma in working adults and the occurrence of absences concluded that once adjusting with comorbid chronic conditions, there was no difference between having asthma and absenteeism [6]. This may suggest that if both the parent and their children have asthma, the parent will lose more working days due to their children's condition. In addition, the annual US economic cost of asthma in 2013 was $81.9 billion, and the annual average cost of asthma, per person, was $3,266 [7]. The financial burden, of individual people with asthma, has increased as the price of inhaled asthma medications have increased in the past several years [7].

The older adult population is increasing worldwide, and a significant percentage has asthma, which is frequently underdiagnosed and has higher morbidity and mortality rates compared with their younger counterparts [8]. Studies show that 30–40% of adults with asthma have their first attack after the 40 years [9], and almost 20% of older adults with asthma receive an improper diagnosis of chronic obstructive pulmonary disease [10].

Asthma self-management education consists of information on asthma, self-monitoring, regular medical follow-ups, a written asthma action plan, instructions on the use of prescribed inhalers, education about recognition of early signs and symptoms of an asthma episode, and appropriate responses and advisement to reduce environmental/allergen exposure as appropriate [11, 12]. Some studies reported better outcomes in asthma with the use of the Asthma Action Plan [13].

Previous studies have shown that asthma education provided in various settings such as health care facilities, schools and communities helped people with asthma and their caregivers to detect the asthma triggers and symptoms in the early stage, as well as prevent asthma attacks and frequent emergency room visits [14, 15]. A study focused on adults home-based asthma education program using Respiratory Therapists, who conducted all the home visits, asthma education, environmental assessments, and implementation of an asthma action plan for participants (mostly Hispanic), revealed improved health outcomes and identified special behaviors that are important such as the frequency of washing the bed linens.

In addition, asthma education programs that are culturally competent are significantly effective in improving asthma-related outcomes such as medication adherence, cost-effectiveness, asthma knowledge, and emergency room visits, especially for ethnic minorities [16, 17]. Two studies showed that culturally competent multifamily asthma treatment and “one-on-one” educational models increased self-efficacy and knowledge of asthma as well as reduced emergency room visits among minorities [18, 19]. Thus, asthma education is recommended for all people with asthma and their families as a significant part of asthma care and self-management [13, 20].

However, multiple factors cause disparities in asthma management/education among ethnic minorities. These factors include low socioeconomic status, lack of accessibility to health care due to transportation issues, inadequate insurance coverage, communication difficulty due to language barriers, insufficient knowledge of asthma care and management, lack of knowledge about asthma triggers, concerns regarding dependency, and side effects of medications in the long term [17, 21–26]. Mainly, the lack of accessible and trained asthma educators, to whom physicians can refer asthma patients, may additionally be a part of the issue. In South Texas, especially in hard-to-reach communities, difficulty in seeing a physician could be due to transportation barriers, which include travel to the medical office or clinic, access to a vehicle, and lack of public transportation. A study from a cross-sectional household survey, conducted by the US Department of Transportation, examined ethnic differences in the burden of travel for health care services, which revealed that African Americans and Latinos had more severe troubles for mobilization compared with Whites [27].

The Texas Asthma Control Program (TACP) was established in 2002 but was discontinued in 2014 due to lack of funds. As of August 2019, the state of Texas was not funded under CDC's National Asthma Control Program [28]; however, during the month of October, we received the news through a conversation with Heather Bartero, MPH (October 2019) that CDC has provided funds to the TACP program. TACP's mission is to decrease asthma morbidity and reduce the social and economic impact of asthma. Along with many partners across the state who have a common vision for asthma management, TACP's goals are to reduce the severity of asthma symptoms and decrease the number of emergency department hospital visits and deaths due to asthma through education and awareness campaigns. As part of the TACP's initiatives in 2007, the program offered seed money in diverse counties, with a higher prevalence of asthma and hospitalizations, to begin an asthma coalition or to develop a project that would focus on improving the management of asthma in the communities.

The Asthma Coalition of Texas was formed by the Texas Department of State Health Services (DSHS) and the coalitions that were initiated by the seed money provided by TACP [29]. Unfortunately, after the closure of the TACP in 2014, many of the coalitions have disappeared, so the TACP program is trying to renew their stakeholders at this moment. Currently, three coalitions (Included in Table 1) are active in the state of Texas, which are The South Texas Asthma Coalition's Pediatric Managing Asthma Through Case Management in Homes, Gulf Coast Asthma Coalition, and Coastal Bend Asthma Initiative. The McAllen Coalition evolved to form the Program on Asthma Research and Education, housed in Texas A&M University School of Public Health [30].

There is still a paucity in studies to identify the distribution of the current asthma education programs and marginalized areas with high needs for asthma education programs. Therefore, the objective of this study is to identify the distribution of asthma education programs that are currently active in Texas and to examine whether there is a geographical disparity between the prevalence of asthma and asthma education programs in the level of the Texas Public Health Regions (PHRs).

## 2. Materials and Methods

### 2.1. Data

This is a descriptive study to identify the distribution of the current asthma education programs and examine whether there is a regional disparity between asthma prevalence and the distribution of asthma education programs in Texas. In this study, adult asthma prevalence data in the PHR level of Texas was obtained from the 2015 Texas Behavioral Risk Factor Surveillance System (BRFSS) Public Use Data File of the Texas DSHS. The prevalence of asthma was weighted to reflect the total adult population by demographic characteristics, based on a complex population-based weighting methodology known as iterative proportional fitting. The total unweighted sample size of adults who answered questions about asthma status was 14,588, and the population-weighted prevalence was estimated based on the total adult population in each region (total 20,203,237).

The total number of adults older than 18 years with asthma was based on the response to the following two survey questions: “Has a doctor, nurse, or other health professionals ever told you that you had asthma?” and “Do you still have asthma?” In a report developed in the spring of 2014 [31], a list of 51 asthma programs was included as active in Texas, which helped us with our research. The contact information and website addresses of the programs were obtained from that report. The research team, in June 2019, searched for asthma programs on the Internet and later contacted those identified programs. Through professional networks and direct phone calls to the program representatives, it was possible to confirm whether the programs were still active. As a result, 24 programs were identified as operational in Texas, and 27 programs were confirmed to be no longer active or duplicated with existing programs. Through the additional search and phone calls, three coalitions were found to be active, leading to the final compendium of 27 asthma education programs included in this study, which are currently active in Texas as of July 22, 2019. Furthermore, we gathered information about each program, including the name of the organization, the name of the education program, the location (address), the type of organization, the target population, and the contact information.

### 2.2. Measure

The Texas DSHS divides the state of Texas into 11 PHRs. These 11 PHRs comprise all regions in Texas, and they are responsible for delivering comprehensive health services to the counties under them [32]. All of the 11 PHRs in Texas were included in this study.

The adult asthma prevalence was measured as the percentage of adults diagnosed with asthma in each PHR in the 2015 Texas BRFSS Public Use Data File. Using the Jenks natural breaks classification method [33], we categorized PHR into four groups: group 1 (5.1–5.2%), group 2 (5.3–7.2%), group 3 (7.3–8.5%), and group 4 (8.6–11.5%).

### 2.3. Statistical and Spatial Analysis

We conducted a descriptive analysis to calculate mean, median, standard deviation (SD), and interquartile range (IQR) of the number of people with asthma, the adult asthma prevalence, the number of asthma education programs per PHR, and the number of population with asthma per program. A correlation test was performed to examine the relationship between adult asthma prevalence and the number of asthma education programs among PHRs using STATA, version 14 (StataCorp LP, College Station, TX, USA). In addition, the Geographic Information System (GIS) program was used to show the distribution of asthma education programs and visually identify the isolated areas for asthma education on the maps. The location of all current asthma education programs was geocoded on the map of Texas, based on latitude and longitude of each program's address. To examine the areas covered by the asthma education programs, we illustrated 50 miles and 70 miles of buffer zones from each program by proximity (multiple ring buffer) functions in GIS. The buffer zones were indicated with different colors. Spatial analysis was conducted using ArcGIS software 10.4 (ESRI, Redlands, CA).

## 3. Results

Twenty-seven asthma education programs, which are currently active in Texas, were obtained for this study (Table 1). Based on the organization type, these programs are identified as health care/hospital systems, educational institutions, independent school districts, other agencies/community collaborations, and coalitions. These organizations provide asthma education to asthmatic patients and their families, school personnel, and health care professionals through their personalized educational curriculums.  Table 2 shows the significant characteristics of all of the asthma programs in Texas.

Table 3 shows the descriptive statistics of 11 PHRs in Texas. We found that there was a large variation in the population with asthma among PHRs, and its range was from 29,428 (PHR 10) to 366,431 (PHR 3). The mean and median percentage of adult asthma was 7.6 and 7.2, respectively. PHR 2 had the highest rate (11.5%), and region 10 had the lowest rate (5.1%). The distribution of the asthma education programs also showed a considerable variation among PHRs in Texas. Among the 27 asthma education programs, the average number of education programs was 2.4 and ranged between 0 and 8. Three regions (PHRs 2, 5, and 9) did not have any education programs, while the other two regions (PHRs 3 and 6) had 15 asthma education programs (55.5% of the total programs). The mean and median numbers of population with asthma per program were 56,271 and 51,825. A big difference was found between the highest (PHR 7/113,721) and the lowest PHRs (PHR 11/18,628). In addition, we found that regions with higher adult asthma prevalence than the average (PHRs 1, 2, 4, 7, and 8) had also above average values in the number of adults with asthma per program.

**Figure 1:**
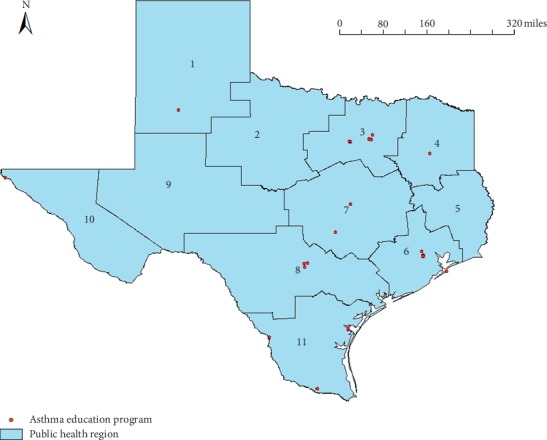
Distribution of asthma education programs in Texas (2019).

Figure 1 demonstrates the distribution of the currently active asthma education programs in Texas. We found that the asthma education programs are located around major cities, including Dallas/Fort Worth (PHR 3), Houston (PHR 6), Austin (PHR 7), and San Antonio (PHR 8), in Texas. This map shows that the locations of the majority of the asthma education programs had a much-skewed distribution, concentrating on some areas, especially major cities. In addition, some of the education programs are in the Texas-Mexico border areas (PHRs 10 and 11). This unbalanced distribution affected a large variation of the population per program among Texas PHRs. Residents in three PHRs (2, 5, and 9) do not have any asthma education programs in their regions. In addition, PHR 7 has two asthma education programs but still the highest number of population with asthma per program (113,721).

**Figure 2:**
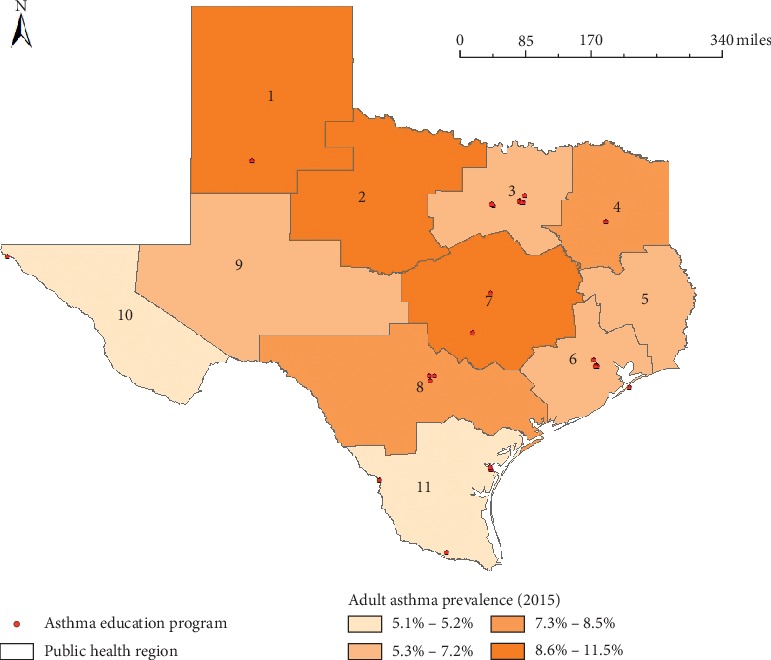
Adult asthma prevalence and education programs in Texas.

The distributions of adult asthma prevalence and asthma education programs are shown in Figure 2. The highest group for adult asthma prevalence included PHRs 1, 2, and 7, while the lowest group had PHRs 10 and 11. The former (*n* = 3) had fewer education programs than the latter (*n* = 4). Therefore, we found that there is a regional imbalance between asthma prevalence and locations of asthma education programs. In particular, PHR 2 is one of the highest regions for the asthma rate; however, there are no asthma education programs, which indicate that people with asthma living in PHR 2 have limited access to the education programs. In addition, the correlation test between asthma prevalence and the number of education programs showed that there was a negative correlation (−0.342), indicating that adult asthma prevalence is associated with the decreased number of asthma education programs. However, this was not statistically significant (*p*=0.304).

**Figure 3:**
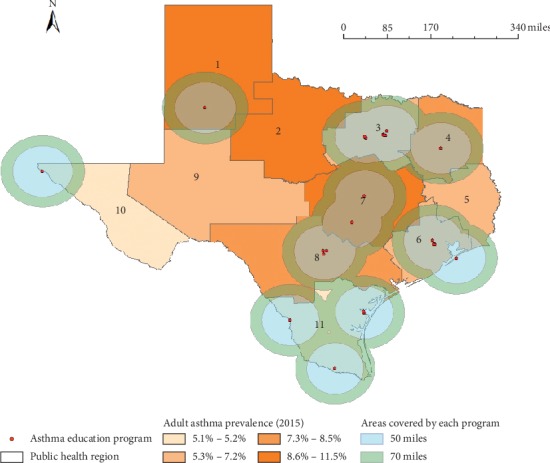
Estimated regions covered by asthma education programs in Texas.

When we displayed the buffer zones of 50 miles or 70 miles, which may be covered by the current asthma education programs (see Figure 3), the marginalized areas for asthma education programs were identified. That is, we found that the active asthma education programs are accessible within 70 miles in most of the eastern and southern areas of Texas. However, the central and the western areas of Texas, particularly most of PHRs 2 and 9, are not covered by the buffer zones of 70 miles from the asthma education programs.

## 4. Discussion

The results of this study showed that there is a lack of asthma coalitions/programs in PHRs 2 and 9 and a high number in PHR 6. There is a need to implement new coalitions in PHRs where the number of adults with asthma is high and no asthma resources are identified. Depending upon the PHR that an adult with asthma lives, if there is an asthma program that could provide education in signs, symptoms, self-management, and asthma triggers, they will benefit greatly from it. It is known that environmental and social barriers for adult's asthma self-management, extreme living conditions, make it difficult or impossible for patients to attend medical visits and adhere to optimal asthma care. Lack of knowledge of these barriers does not help health providers to create tailored, personal, and empathetic approaches to asthma management. Depending on their economic situation, adults with asthma might have high morbidity, many hospitalizations and emergency department visits, other comorbidities, and significant barriers including difficulty with transportation to regular outpatient visits [34]. To adequately manage asthma, those suffering from it require an understanding of the nature of asthma, their asthma triggers and how to decrease and prevent them, the basic principles of the treatment protocol, and how to evaluate its control and the characteristics related to their case. Additionally, teaching self-management skills to people with asthma will help them manage their asthma effectively [35–37]. A study reported that having a controlled asthma and good health status (use of daily inhaled corticosteroids) were associated with significantly fewer emergency department visits and better asthma control [38, 39].

Health care professionals play an essential role in the dissemination of education to people with asthma; however, due to limited resources and time constraints, it may not seem possible. It is imperative to provide or make available information on asthma coalitions and programs to health care professionals as it can help them provide education to patients and their caregivers at convenient places. The knowledge that the patients acquire will be useful to help identify the triggers of asthma, recognize the signs and symptoms of asthma, and adhere to treatment [40–43]. Therefore, developing a partnership between physicians, patients, and programs/coalitions to provide asthma education can be a successful strategy to improve self-management as well as preventive measures regarding asthma triggers [44].

Unfortunately, information about coalitions and programs that offer asthma education is not easily obtainable by everyone, and the availability of adequately trained educators/professionals in asthma is vital. The absence of asthma education is related to factors such as language barriers, lack of resource knowledge, nonintegration of asthma education by physicians into current practice, and lack of knowledge of those programs or coalitions in their cities. Patients are sometimes reluctant to attend educational programs, even when available at minimal or no cost. Participating in those asthma programs may be difficult due to lack of transportation, inadequate motivation, lack of time, or the perception that they do not need this intervention [35]. Asthma education programs that are culturally competent are significantly effective in improving asthma-related outcomes, especially for ethnic minorities and hard-to-reach communities. To develop a culturally competent and successful intervention, health care providers should identify culture-based specific characteristics reflecting the cultural differences and tailor interventions based on those characteristics [17]. There have been studies that discussed alternative methods such as traveling asthma programs and telehealth platforms [45, 46], and these methods can also be considered to develop continuing education programs to overcome these obstacles.

This study demonstrates the distribution of the current asthma education programs in Texas and provides a visual representation of the marginalized areas for asthma education programs. The information presented in this study is needed at many levels, such as academic, government, state, nonprofit organizations, and communities. During the existence of TACP, information regarding asthma was readily available from the Texas DSHS. Since its closure in 2014, current asthma information in Texas and its counties are scarce, outdated, and only researchers, working with asthma in affected communities might have the current statistics; however, now that it is funded again, we hope to have the information that is needed to develop new asthma programs where they are needed. This study provides information about the available coalitions and programs functioning as of July 2019, with their contact information in the state of Texas.

However, this study has several limitations. First, the process used for data collection had several limitations identifying programs because some of them were not available through the website search or embedded in larger chronic disease initiatives. Therefore, there are additional asthma program resources available in the state of Texas, which are difficult to identify and access. These programs require improved presence through the Internet. Among calls to the 51 programs, only 23 representatives followed up and provided detailed information about their programs. Secondly, the information about the age range and number of participants attending their programs was not included in this study. Thirdly, we used the level of PHRs as boundaries considering the data availability for asthma prevalence. In addition, we did not have the data about the estimated number of population with asthma within each buffer; therefore, it was hard to see how many populations are covered by current asthma education programs. Future research needs to use smaller boundaries, such as counties and census-tract level, to identify the regions with high demands for asthma education. Fourthly, we covered only adult asthma prevalence in this study due to data availability. Child asthma prevalence will need to be included in further studies. Lastly, only on-site education programs were considered in this study, although there might be other types of educational programs like online-based and telehealth education. Therefore, further studies should include these kinds of educational programs.

## 5. Conclusions

We concluded that there is a regional disparity between adult asthma prevalence and the distribution of asthma education programs in Texas. This study identified the marginalized regions in Texas with high demands for asthma education programs. The findings could help policymakers, and health care professionals to enhance opportunities to develop asthma education programs. Diverse venues can be identified for isolated areas for asthma education programs and prioritize these regions for funding innovative programs. The government, foundations, and other health care agencies could consider providing funds for initiatives that could reduce geographic disparity for asthma education programs.

## Figures and Tables

**Table 1:** Compendium of active asthma education/management programs in Texas (2019).

PHR	Organization name	Organization type	Education program	Address/County	Target population
1	University Medical Center (UMC)-Lubbock	Educational institution	Camp zone	3502 22nd Street, Lubbock, TX 79410/Lubbock County	Asthmatic children (aged 7–11)
				806-392-6128	

3	Asthma Chasers/Positive Breathing	Other agencies/community collaborations	Asthma chasers/positive breathing	P.O. Box 742422, Dallas, TX 75374,/Dallas County	Parents, children and caregivers
				469-245-7994	

3	Baylor University Medical Center at Dallas—Martha Foster Lung Care Center	Educational institution	Dallas independent school district program	3500 Gaston Avenue, Dallas, TX, 75246/Dallas County	School nurses, nurse assistants and other school personal, adult and pediatric patients
				214-820-9774	

3	Children's Medical Center of Dallas	Health care/hospital system	Asthma management program (the health and wellness alliance for children)	1935 Medical District Dr., Dallas, TX 75235/Dallas County	Pediatric patients
				214-456-7000	

3	Cook Children's Pulmonology	Health care/hospital system	Asthma education	1500 Cooper St., Fort Worth, TX 76104/Tarrant County	Children with asthma and their families
				254-935-4981	

3	Environmental Protection Agency (EPA) Region-6	Other agencies/community collaborations	Asthma	1445 Ross Ave., Suite 1200, Dallas, TX 75202/Dallas County	Public/community
				214-665-6663	

3	John Peter Smith Hospital	Health care/hospital system	School based care (SBC)—chronic care model	1500 S Main St., Fort Worth, TX, 76104/Tarrant County	Pediatric patients
				81t7-702-3431	

3	Parkland Health & Hospital System	Health care/hospital system	Better breathing program	5201 Harry Hines Blvd., Dallas, TX 75235/Dallas County	Pediatric patients
				214-590-8000	

3	University of North Texas Health Science Center (in collaboration with Fort Worth Independent School District)	Educational institution	Asthma 411: A collaborative asthma initiative to improve community health	3500 Camp Bowie Blvd, Fort Worth, TX 76107/Tarrant County	School children
				817-735-5098	

4	The University of Texas Health Northeast	Educational institution	Breath of life – Mobile	11937 US HWY 271, Tyler, TX 75708/Smith County	School children
				903-877-8925	

6	American Lung Association of the Southwest States (Houston)	Other agencies/community collaborations	ALA initiatives	2030 North Loop West, Suite 250, Houston, TX 77018/Harris County	Community, health workers and schools
				713-629-5864	

6	Baylor College of Medicine-Environmental Health Service	Educational institution	BCM environmental health service	1 Baylor Plaza, Houston, TX 77030/Harris County	Healthcare professionals and the public
				713-798-1082	
				214-820-9774	

6	Gulf Coast Asthma Coalition	Coalition	Gulf coast asthma coalition	8000 N. Stadium, Houston, TX 77063/Harris County	General population
				832-393-5154	

	11 Coastal Bend Asthma Initiative	Coalition	Coastal bend asthma initiative	209 North Water Street, Corpus Christi, TX 78401/Neuces County	Mainly children with asthma
				361-694-6547	

6	Texas Children's Health Insurance Plan	Other agencies/community collaborations	TCHIP asthma program	2450 Holocombe Blvd #34l, Houston, TX 77021/Harris County	Pediatric patients
				832-824-1000	

6	Texas Children's Hospital	Health care/hospital system	NA	6621 Fannin St, Houston, TX 77030/Harris County	Pediatric patients
				832-822-3315	

6	The University of Texas Health Science Center at Houston	Educational institution	NA	6431 Fannin St., Houston, TX, 77030/Harris County	Pediatric/Adolescent patients
				713-500-5732	

6	University of Texas Medical Branch Hospital	Educational institution	UTMB Children's asthma program	301 University Boulevard, Galveston, TX 77555/Galveston County	Pediatric patients
				281-221-8853	

7	McLane Children's Scott and White Children's Hospital	Health care/hospital system	McLane Children's scott & white asthma outreach program	1901 SW H K Dodgen Loop, Temple, TX 76502/Bell County	Patients, schools and communities
				254-935-4981	

7	Seton Healthcare Family	Health care/hospital system	Seton asthma center	5555 N. Lamar Blvd., Austin, TX 78751/Travis County	Pediatric patients and their families
				512-324-5909	

8	Northeast Independent School District	Independent school district	Asthma awareness education program	8961 Tesoro Dr., San Antonio, TX 78217/Bexar County	School children an staff
				210-356-9247	

8	South Texas Asthma Coalition's Pediatric Managing Asthma through Case Management in Homes (PMATCH)	Coalition	PMATCH	8207 Callaghan Road, #140 San Antonio, Texas 78230/Bexar County	Children with asthma and their families
				210-602-3113	

8	University Health System (University Hospital)—Respiratory Care Department	Educational institution	Asthma education	701 S Zarzamora St, San Antonio, TX 78207/Bexar County	Adult and pediatric patients
				210-358-2798	

10	Mid Rio Grande Border Area Health Education Center (MRGB AHEC)	Educational institution	Annual asthma education and screening program	1505 Calle Del Norte, Suite 430, Laredo, TX 78041/Webb County	Adults and children
				956-712-0037	

10	The Hospitals of Providence Memorial Campus	Health care/hospital system	Community health program	2001 N Oregon, El Paso, Texas 79902/El Paso County	Pediatric patients
				915-577-6992	

11	Driscoll Children's Hospital—Coastal Bend Asthma Initiative	Health care/Hospital system	Camp easy breathers	3533 S Alameda St, Corpus Christi, TX 78411/Nueces County	Pediatric patients
				361-694-4580	

11	Texas A&M School of Public Health	Other agencies/community collaborations	PARE	2101 McColl Rd, McAllen/Hidalgo County	Families with asthma
				(956) 668–6311	

**Table 2:** Characteristics of asthma education programs in Texas (2019).

Organization type	*N* (total = 27)	Target population	Setting of delivery
Health care/hospital systems	9	Children with asthma and their families, the community and schools	Clinic based, school based, camp based, and community based
Educational institution	9	Health care professionals, the public, school nurses and school staff, and school children diagnosed with asthma	Clinic based, school based, camp based, community based
Independent school district	1	School children and school staff	School based
Other agencies/community collaborations	5	Parents, caregivers and children, the public, community health workers (CHWs), and schools	Clinic based and community based
Coalitions	3	Community, children with asthma, and their families	Community based

**Table 3:** Descriptive statistics of 11 public health regions in Texas.

	Number of adult population with asthma (2015)	Adult asthma prevalence (2015) (%)	Asthma education programs (2019)	
			Number	People with asthma per program
Total public health regions (PHRs)				
Mean	133,303	7.6	2.4	56,271
SD	116,313	1.96	2.67	27,446
Median	73,923	7.2	1	51,825
IQR	152,213	2.3	3	25,533
Individual public health regions (PHRs)				
Region 1	73,923	10.4	1	73,923
Region 2	52,068	11.5	0	—
Region 3	366,431	7.0	8	45,804
Region 4	65,016	8.4	1	65,016
Region 5	41,594	7.2	0	-
Region 6	327,374	6.8	7	46,768
Region 7	227,442	9.8	2	113,721
Region 8	170,646	8.5	3	56,882
Region 9	37,901	6.9	0	—
Region 10	29,428	5.1	1	29,428
Region 11	74,512	5.2	4	18,628

*Note.* Asthma data are population estimate after weighting for the population; SD = standard deviation; IQR = interquartile range.

## Data Availability

The data used to support the findings of this study are available from the corresponding author upon request.
